# TP53 Mutational Status-Based Genomic Signature for Prognosis and Predicting Therapeutic Response in Pancreatic Cancer

**DOI:** 10.3389/fcell.2021.665265

**Published:** 2021-05-26

**Authors:** Feng Zhang, Wenhui Zhong, Honghao Li, Kaijun Huang, Min Yu, Yubin Liu

**Affiliations:** ^1^Department of General Surgery, Guangdong Provincial People’s Hospital, Guangdong Academy of Medical Sciences, Guangzhou, China; ^2^Shantou University Medical College, Shantou, China; ^3^The Sixth Affiliated Hospital, Sun Yat-sen University Guangdong Gastrointestinal Hospital, Guangzhou, China

**Keywords:** TP53 mutation, pancreatic cancer, signature, prognosis, therapeutic responses

## Abstract

TP53 mutation is a critical driver mutation that affects the carcinogenesis and prognosis of patients with pancreatic cancer (PC). Currently, there is no driver mutation-derived signature based on TP53 mutational status for prognosis and predicting therapeutic response in PC. In the present study, we characterized the TP53 mutational phenotypes in multiple patient cohorts and developed a prognostic TP53-associated signature based on differentially expressed genes between PC samples with mutated TP53 and wild-type TP53. Comprehensive investigations were carried out in prognostic stratification, genetic variation, immune cell infiltration, and efficacy prediction of chemotherapy and targeted therapy. We found that TP53 mutation commonly occurred as a survival-related driver mutation in PC. In total, 1,154 differentially expressed genes were found between two distinct TP53 mutational phenotypes. A five-gene TP53-associated signature was constructed in The Cancer Genome Atlas (TCGA) cohort by least absolute shrinkage and selection operator (LASSO)–Cox analysis and proven to be a robust prognostic predictor, which performed well in three independent Gene Expression Omnibus (GEO) validating cohorts. Remarkably, patients in the low-risk group were characterized with decreased tumor mutation burden and activity of immunity, with favorable prognosis. Higher fractions of macrophages M0 and impaired CD8 + T cells were observed in patients in the high-risk group, suggesting immunosuppression with poor survival. Patients in the high-risk group also demonstrated enhanced response to specific chemotherapeutic agents, including gemcitabine and paclitaxel. Several targeted inhibitors, like histamine receptor inhibitor, were screened out as promising drugs for PC treatment. Collectively, the TP53-associated signature is a novel prognostic biomarker and predictive indicator of PC. The signature could contribute to optimizing prognostic stratification and guide effective PC treatments.

## Introduction

Pancreatic cancer (PC) is an aggressive and lethal malignancy with a dismal 5 years survival rate of 4% and 47,050 cancer-related deaths in 2020 (Klint et al., 2010; Siegel et al., 2020). Because of the limited treatment options and deficiency of robust biomarkers for early stage screening, 80% of patients with PC were typically diagnosed in advanced stage and not candidates for surgical intervention (Sohn et al., 2000; Winter et al., 2006). Moreover, the survival of PC has not significantly improved even for those who received surgery at the early stage (Kasumova et al., 2018; Strobel et al., 2019). Currently, several targeted drugs have emerged as potentially effective treatments for PC; however, the limitation is that only a small subset of patients with specified characteristics may benefit from these targeted approaches (Kleeff et al., 2016; Osmani et al., 2018; Zhu et al., 2018). As a consequence, there is an urgent need to better stratify prognosis and develop more suitable therapeutic strategies for patients with PC.

The tumor suppressor gene TP53 is one of the frequent pan-cancer mutated genes, linked to unfavorable prognosis in multiple cancers and more than 500 million deaths (Kandoth et al., 2013). Functionally activated by a series of stress stimuli, wild-type TP53 protein exquisitely manages complex transcriptional processes involved in apoptosis and anti-proliferation (Kastenhuber and Lowe, 2017). Mutation of TP53 occupies one of the identified major driver mutations presented in the complex mutational landscape of PC (Jones et al., 2008; Makohon-Moore and Iacobuzio-Donahue, 2016). Occurring in about 70% of examples, TP53 mutation often leads to an oncogenic process and is associated with aggressive and metastatic phenotypes (Makohon-Moore and Iacobuzio-Donahue, 2016; Hashimoto et al., 2019). The tumor-suppressive effect of TP53 and the prevalence of TP53 mutation have encouraged the development of precise therapy targeting TP53 network in cancers. For example, MK-1775, SGT-53, Alisertib, and AMG900 are several promising anti-PC drugs that target TP53 and tested in ongoing clinical trials. Interestingly, recent studies depicted that TP53 mutational status is closely associated with various antitumor immune responses (Swidnicka-Siergiejko et al., 2017; Hashimoto et al., 2019). The regulatory effect on immune response of TP53 mutation has been proposed (Butin-Israeli et al., 2019; Blagih et al., 2020). P53 induction was associated with peptide processing and major histocompatibility complex (MHC)-I surface expression, thus it might prevent the cytotoxic T lymphocytes (CTLs) from killing tumor cells (Wang et al., 2013).

Previous studies have assessed the prognostic value of pancreatic driver mutations. However, to date, few robust and reliable driver mutation-related biomarkers were identified to predict prognosis and therapeutic response. Here, we present a comprehensive study to describe the mutational landscape of PC and the difference of TP53 mutational status and then constructed a TP53-associated prognostic signature in The Cancer Genome Atlas (TCGA) cohort. External validation was performed in the GSE28735, GSE62452, and GSE78229 cohorts to illustrate its prognostic efficacy. Furthermore, the associations of TP53 mutational signature with genetic mutation, tumor microenvironment, and multidimensional therapeutic application were investigated. This novel model could be used for screening, prognostic assessment, and treatment approaches in PC.

## Materials and Methods

### Data Source and Processing

The public VarScan2 somatic mutations, corresponding transcriptional expressions, and full clinical annotation of PC patients were obtained from TCGA^1^ and Gene Expression Omnibus (GEO)^2^ databases. In total, 151 patients from TCGA-pancreatic adenocarcinoma (PAAD), 42 patients from GSE28735, 66 patients from GSE62452, and 49 patients from GSE78229 cohorts were collected for analysis. For the transcriptional profile in TCGA, transcripts per kilobase million (TPM) values or log2 transformations were performed in the gene expression data. In the GEO microarray data, batch effects were eliminated *via* the combat algorithm of “sva” package, and then data normalization was conducted by “limma” package (Ritchie et al., 2015). The downloaded data were utilized according to TCGA and GEO data access requirements. All mutation data, gene expression profile matrix, and clinical feature data of PC are publicly available.

### Identification of Differentially Expressed Genes

To identify the differentially expressed genes (DEGs) based on different TP53 mutational statuses, we classified patients into two TP53 mutational phenotypes. Under the threshold of | log2 fold change| (log^2^FC) ≥ 1 and false discovery rate (FDR) < 0.01, the “limma” R package was applied to determine DEGs between 82 PC samples with mutated TP53 and 69 PC samples with wild-type TP53 in TCGA cohort (Ritchie et al., 2015).

### Construction and Validation of a TP53-Associated Prognostic Signature

A total of 151 PC samples with complete TP53 mutation data, gene expression profile, and survival data in TCGA cohort were subjected to analyses. Univariate Cox regression analysis was performed among DEGs *via* “survival” R package to figure out significantly prognostic DEGs associated with overall survival (OS). Next, the key prognostic DEGs were screened out, and the collinear problem was removed by the analysis of least absolute shrinkage and selection operator (LASSO). Multivariate Cox regression analysis was implemented to figure out the independent prognostic DEGs. We applied LASSO–penalized Cox regression analysis to further narrow the OS-related DEGs and construct a five-gene signature panel in TCGA cohort. The signature risk scores were calculated according to the sum of the multivariable regression coefficients multiplied by the expression level of each model gene. Defined by the cutoff equal to the median risk score, samples were classified into the high- and low-risk groups. Survival was measured utilizing the Kaplan–Meier method and log-rank test.

### Estimation of Immune Cell Infiltration

To characterize the tumor-infiltrating immune cell fraction in PC, gene expression profile was normalized and written by standard annotation file, subsequently uploaded to the Cell type Identification by Estimating Relative Subsets of RNA Transcripts (CIBERSORT) approach combined with the LM22 gene signature (Newman et al., 2015). Then, we quantitatively evaluated the abundance of 22 types of immune cells between high- and low-risk groups based on the signature. We access the marker gene set for infiltrated immune cell types offered by Bindea et al. (2013).

### Tumor Immune Dysfunction and Exclusion

The Tumor Immune Dysfunction and Exclusion (TIDE)^3^ is a data-driven Web platform that integrates large-scale omics data of over 33,000 cases from 188 cohorts, 998 tumor samples from 12 immune checkpoint blockade (ICB) clinical studies, and eight clustered regularly interspaced short palindromic repeats (CRISPR) screens. The TIDE could contribute to hypothesis generation and immunological biomarker optimization (Fu et al., 2020). In the present study, we used the TIDE to evaluate the impact of expression of the five genes on T cell dysfunction, immune-suppressive rejection score, and therapeutic response of ICB.

### Prediction of Chemotherapeutic and Targeted-Therapeutic Response

Individual chemotherapeutic response was estimated according to the Genomics of Drug Sensitivity in Cancer (GDSC)^4^, a public pharmacological Web portal accessible to predictive sensitivity of 138 common chemotherapeutic agents. We used the ridge regression to estimate the half-maximal inhibitory concentration (IC_50_) and implement 10-fold cross-validation *via* “pRRophetic” R package. Then, the Connectivity Map (CMap) database was used to search for potential inhibitors or compounds that targeted TP53-associated signature (*p* < 0.05). Mode-of-action (MoA) analysis was performed to figure out the potential mechanism of those candidate drugs (Lamb et al., 2006).

### Statistical Analysis

The statistical significance of mean value of variables between two groups was calculated by unpaired Student’s *t*-tests. We adopted two-sided Fisher’s exact tests to analyze contingency tables. As for the correlation between risk score and patients’ outcome, the cutoff value of each subgroup was determined using the “survminer” R package. The survival curves were generated *via* Kaplan–Meier method, and the significance of survival differences was determined using the log-rank test. Univariate and multivariate Cox proportional hazard models were utilized to calculate the hazard ratios of variables and determine independent prognostic factors. LASSO analysis was carried out to get rid of the collinear problem and screen important prognostic genes. The predictive accuracy of the prognostic models was quantified through time-dependent and -independent receiver operating characteristic (ROC) curves. The waterfall function of “maftools” R package was used to describe the mutation landscape in patients with high and low risk in TCGA-PAAD cohort. Correlation coefficients between risk score and tumor-infiltrating immune cells were computed using Spearman and distance correlation analyses. “pRRophetic” R package was implemented for chemotherapeutic response prediction. All data processing was performed in R software 3.6.2. All statistical significance was set at *p* < 0.05.

## Results

### Phenotypes Based on TP53 Mutational Status in Pancreatic Cancer

**FIGURE 1:**
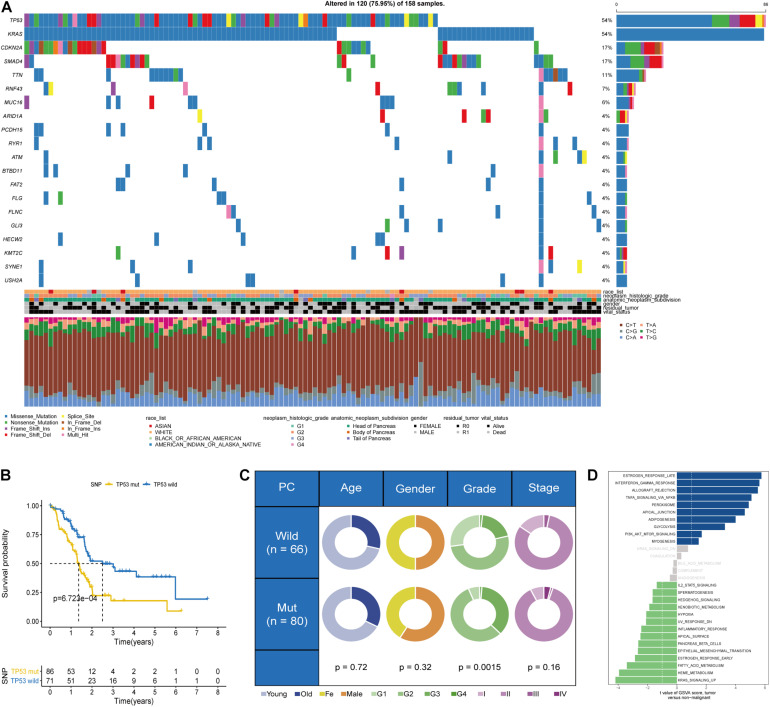
Somatic mutational phenotypes based on the TP53 mutational status in The Cancer Genome Atlas (TCGA) dataset. **(A)** Genomic mutational landscape of pancreatic cancer (PC) in TCGA dataset. **(B)** Kaplan–Meier survival analysis showing that overall survival (OS) was significantly worse in PC patients with TP53 mutation than those without TP53 mutation. **(C)** Pie charts showing the chi-square test of clinicopathological factors in PC tumor samples. **(D)** Difference in the pathway activities scored by Gene Set Variation Analysis (GSVA) between PC patients with and without TP53 mutation.

Data on somatic mutational variations in TCGA cohort were applied to elucidate the mutational landscape of PC and evaluate 20 of the most important gene mutations. In PC, TP53 mutation is one of the frequent somatic mutational types; among them, missense mutation is the most common aberration (Figure 1A). A significant association was observed between TP53 mutational status and prognosis in a deleterious direction (*p* = 6.723e-04, log-rank test), indicating that PC patients with TP53 mutation had worse prognosis than patients without TP53 mutation (Figure 1B). The chi-square test was performed to evaluate the correlation between the TP53 mutational phenotypes and clinicopathological factors. The results indicated that PC patients with TP53 mutation exhibited higher grade of PC than patients without TP53 mutation (*p* = 0.0015; Figure 1C). The single-sample Gene Set Variation Analysis (ssGSVA) method revealed enriched pathways between PC patients in various TP53 mutational subtypes. The direct comparison of the TP53 mutation group vs. the TP53 wild-type group revealed estrogen response and KRAS signaling as the top enriched pathways in PC (Figure 1D).

### Identification of Differentially Expressed Genes and Construction of a TP53-Associated Signature

**FIGURE 2:**
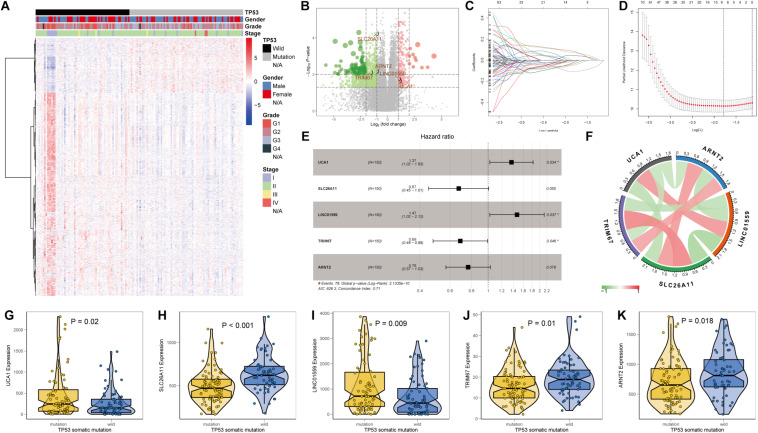
Identification of differentially expressed genes (DEGs) and construction of a TP53-associated signature. **(A)** Heat map and **(B)** volcano plot of DEGs between pancreatic cancer (PC) samples with and without TP53 mutation. Five genes enrolled in the signature were emphasized. **(C)** Least absolute shrinkage and selection operator (LASSO) coefficient profiles of 495 genes. **(D)** Five prognostic genes obtained from LASSO regression with 10-fold cross-validation using minimum lambda value. **(E)** Forest plot, **(F)** mutual correlations, and **(G–K)** relative mRNA expressions of urothelial carcinoma-associated 1 (UCA1), solute carrier family 26 member 11 (SLC26A11), long intergenic non-protein coding RNA 1559 (LINC01559), tripartite motif-containing 67 (TRIM67), and aryl hydrocarbon receptor nuclear translocator 2 (ARNT2).

In view of the significant association between TP53 mutational status and prognosis of PC patients, we aimed to construct a robust prognostic signature based on the DEGs between PC samples with and without TP53 mutation. Differential transcriptional expression analysis was performed using the limma package and meeting the standard of log^2^FC ≥ 1 and FDR < 0.01. In total, 245 upregulated genes and 909 downregulated genes were identified (Figures 2A,B and Supplementary Table 1). Univariate Cox regression analysis indicated that 492 genes were significantly correlated with prognosis of PC patients (*p* < 0.05). Then, those prognostic genes were subjected to the LASSO and multivariable Cox regression analysis (Figures 2C,D). Finally, a TP53-associated signature was constructed based on five genes. Risk score = Exp_*UCA*__1_^∗^ 0.314 − Exp_*SLC*__2__6A__11_^∗^ 0.395 + Exp_*LINC*__01559_^∗^ 0.387 − Exp_*TRIM*__67_^∗^ 0.374 − Exp_*ARNT*__2_^∗^ 0.269 (Figure 2E). Within the signature, solute carrier family 26 member 11 (SLC26A11), tripartite motif-containing 67 (TRIM67), and aryl hydrocarbon receptor nuclear translocator 2 (ARNT2) were downregulated in TP53-mutated PC and positively correlated with each other, while the expressions of urothelial carcinoma-associated 1 (UCA1) and long intergenic non-protein-coding RNA 1559 (LINC01559) were upregulated and positively correlated with each other (*p* < 0.05; Figures 2F–K and Supplementary Table 2). Then, we calculated individual risk score and categorized them into high- or low-risk groups according to the optimal cutoff point in TCGA cohort.

### Evaluation and Validation of the Prognostic TP53-Associated Signature in the Cancer Genome Atlas and Gene Expression Omnibus Cohorts

**FIGURE 3:**
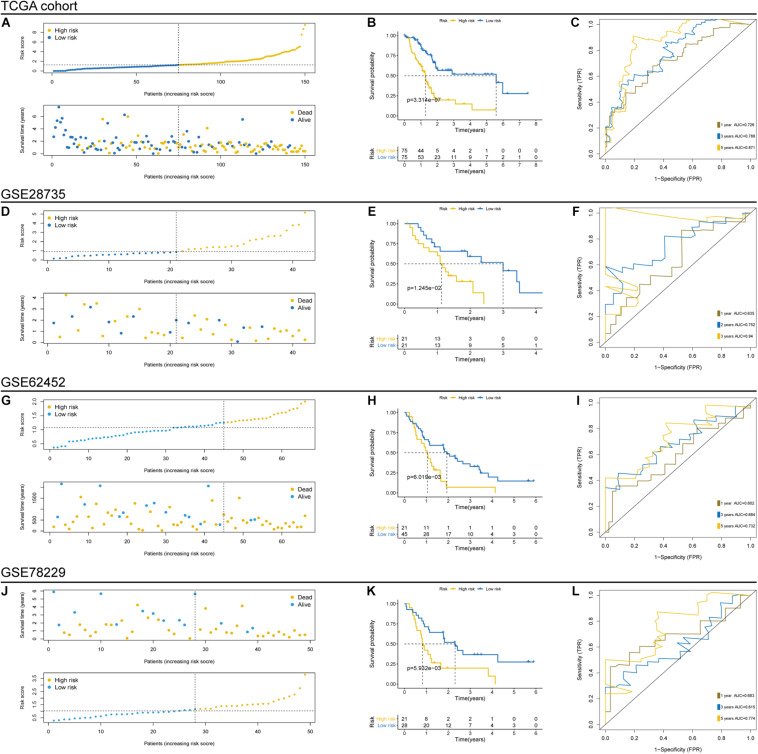
Prognostic evaluation and independent validation of the TP53-associated signature in The Cancer Genome Atlas (TCGA) and Gene Expression Omnibus (GEO) cohorts. Risk score arrangement, survival status, Kaplan–Meier and time-dependent receiver operating characteristic (ROC) analyses in TCGA **(A–C)**, GSE28735 **(D–F)**, GSE62452 **(G–I)**, and GSE78229 **(J–L)** cohorts.

To evaluate the prognostic ability and robustness of the aforementioned TP53-associated signature, its performance was assessed in TCGA and three independent GEO cohorts, including GSE28735, GSE62452, and GSE78229 cohorts. The individual risk score and survival status of patients in the cohorts were shown in Figures 3A,D,G,J. Survival analysis in TCGA cohort indicated that patients in the high-risk group were significantly associated with poor OS (*p* < 0.0001, log-rank test; Figure 3B). The area under the time-dependent ROC curve of the signature was 0.726 at 1 year, 0.788 at 3 years, and 0.871 at 5 years (Figure 3C). Moreover, the TP53-associated signature had well above AUC values compared with the TP53 mutation and clinicopathological factors (Supplementary Figure 1A). Consistent with the performance for OS prediction in TCGA cohort, we found that the TP53-associated signature also worked well in external GEO cohorts, where patients in the high-risk group had unfavorable OS (GSE28735, *p* = 0.0125, Figure 3E; GSE62452, *p* = 0.0060, Figure 3H; GSE78229, *p* = 0.0059, Figure 3K). Moreover, the high accuracy of the signature remained stable in the independent cohorts (GSE28735, Figure 3F; GSE62452, Figure 3I; and GSE78229, Figure 3L). Conditional survival analysis described the probability of achieving 5 years survival in 307 patients from combined TCGA and GEO cohorts increased from 17 to 25, 43, 56, and 72% per additional year survived (i.e., 1, 2, 3, and 4 years, respectively; Supplementary Figure 1B).

### Independent Prognostic Value of the TP53-Associated Signature and Its Correlation With Clinicopathological Characteristics

**FIGURE 4:**
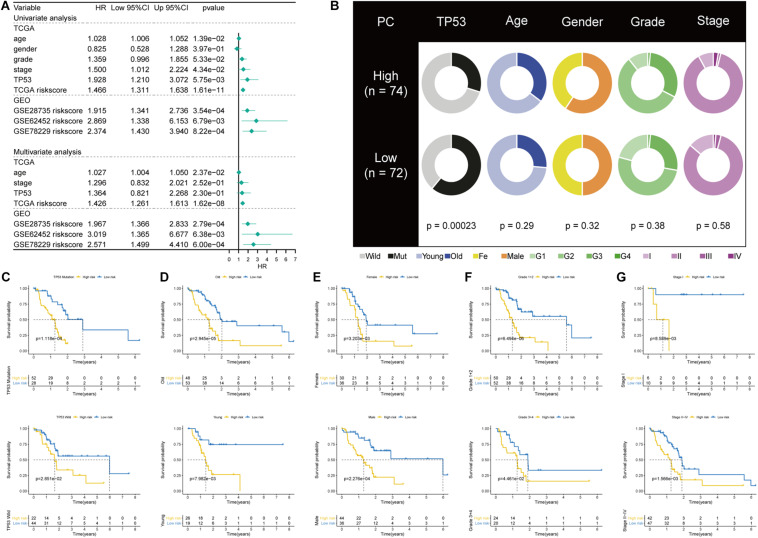
Correlations with clinicopathological characteristics and prognostic independence of the TP53-associated signature. **(A)** Univariate and multivariate regression analyses of the TP53-associated signature and other clinicopathological factors. **(B)** Pie charts showing the chi-square test of clinicopathological factors in pancreatic cancer (PC) samples from The Cancer Genome Atlas (TCGA) cohort based on the TP53-associated signature. **(C–G)** Kaplan–Meier survival analysis of **(C)** TP53 mutation and wild-type subgroup, **(D)** age > 60 and age < 60 subgroup, **(E)** male and female subgroup, **(F)** grades 1 + 2 and 3 + 4 subgroup, **(G)** TNM stage I and II–IV subgroup.

Univariate and multivariate regression analyses showed that the prognostic power of the TP53-associated signature for the OS of PC patients is independent of clinicopathological factors in TCGA cohort (Figure 4A). Figure 4B showed the comparison of clinicopathological factors between high- and low-risk patients. Analysis in the GSE28375, GSE62452, and GSE78229 cohorts also validated that the TP53-associatd signature is an independent prognostic factor. Besides, we performed subgroup survival analysis and risk stratification in patients with varied TP53 mutational statuses, ages, genders, grades, and stages of tumors. Importantly, the TP53-associated signature can also serve as a promising prognostic marker to predict OS in stratified subgroups of patients with PC in TCGA cohort, including TP53 mutation and TP53 wild-type subgroup (*p* = 0.0001 and *p* = 0.02851, respectively; Figure 4C), age > 60 and age < 60 (*p* < 0.0001 and *p* = 0.0079, respectively; Figure 4D), male and female gender (*p* = 0.0002 and *p* = 0.0032, respectively; Figure 4E), grades 1 + 2 and 3 + 4 (*p* < 0.0001 and *p* = 0.0446, respectively; Figure 4F), TNM stages I and II–IV (*p* = 0.0086 and *p* = 0.0156, respectively; Figure 4G). These results demonstrated that the TP53-associated signature is an independent prognostic biomarker.

### Mutational Landscape Based on TP53-Associated Signature

**FIGURE 5:**
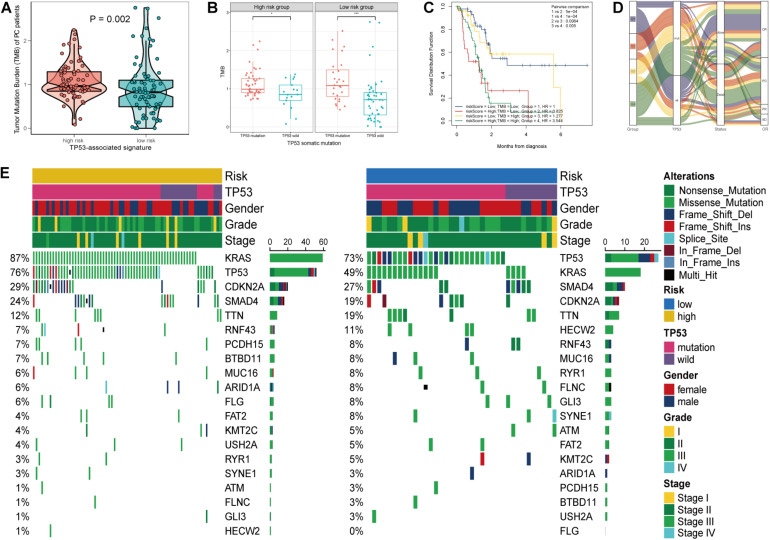
Mutational landscape of the TP53-associated signature. **(A)** Significant difference of tumor mutation burden (TMB) between patients of high- and low-risk group. **(B)** Differential TMB between TP53 mutation and TP53 wild-type pancreatic cancer (PC) based on TP53-associated signature. **(C)** Survival of patients with PC based on the TMB and risk scores. **(D)** Sankey alluvium showing dynamic correlation of patients with PC. **(E)** Waterfall plot of somatic mutation displayed mutational landscape correlated with the signature.

As PC is a malignant disease characterized with highly somatic mutations, we next investigated the association between tumor mutation burden (TMB) and TP53-asociated signature. Patients in the high-risk group and those with TP53 mutation displayed high TMB level (*p* = 0.002, Figure 5A; *p* < 0.001, Figure 5B). We found that the risk score and TMB are significantly correlated with OS (Figure 5C). Associations among the risk score, TMB, TP53 mutational status, survival status, and overall response were demonstrated in Figure 5D. Furthermore, the mutational landscape based on this mutational signature was depicted, and we found that KRAS mutation markedly increased in the high-risk group (Figure 5E). The above results demonstrated the stratified ability of the TP53-associated signature in predicting tumor malignancy.

### Characterization of Immune Cell Infiltration in Distinct TP53 Mutation-Associated Risk Phenotypes

**FIGURE 6:**
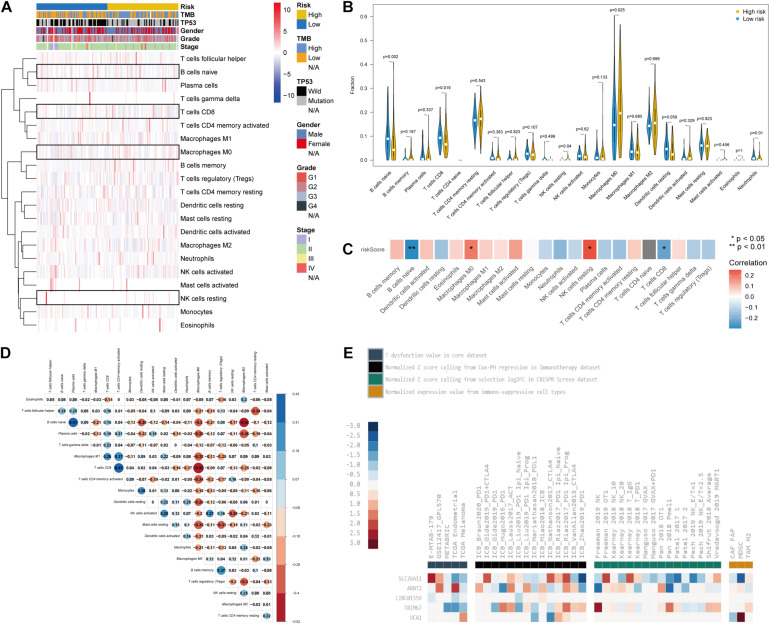
The characteristics of immune cell infiltration and correlation with the TP53-associated signature. **(A)** Immune cell heatmap and clinicopathological characteristics of two phenotypes defined by TP53-associated signature. Four kinds of immune cells were highlighted with boxes. **(B)** Violin plots reflecting the differential composition of 24 types of tumor-infiltrating immune cells. **(C)** Correlation matrix visualizing the relationship between tumor-infiltrating immune cells and risk scores based on the TP53-associated signature. **(D)** Mutual correlations between 24 types of tumor-infiltrating immune cells. **(E)** The role of the five genes in T cell dysfunction, prognosis after immune checkpoint blockade (ICB) therapy, and immunosuppressive T cell rejection.

Recently, several studies emphasized that TP53 mutation status may trigger immune responses and be used as a predictor of immunotherapy in cancers (Dong et al., 2017; Skoulidis et al., 2018). Therefore, we explored the characterization of immune cell infiltration in PC *via* CIBERSORT. Our results showed the difference of tumor-infiltrating immune cells between patients in the high- and low-risk groups (Figure 6A). The variations identified in the immune landscape promoted us to gain insight into the intrinsic traits of individual characteristics. We further investigated the differential composition and association of tumor-infiltrating immune cells by CIBERSORT. Patients in the high-risk group had significantly higher levels of Macrophages M0 and resting natural killer (NK) cells but lower levels of naive B cells and CD8^+^ T cells (*p* < 0.05; Figure 6B). TP53-associated signature was found to be positively correlated with Macrophages M0 and resting NK cells and negatively correlated with naive B cells and CD8^+^ T cells (*p* < 0.05; Figure 6C). Differential and correlated analyses showed consistence in the immunologic characterization. Weak to moderate correlations existed between different types of tumor-infiltrating immune cells (Figure 6D). The immunosuppressive role of the five genes included in the signature was demonstrated by the TIDE database, which involved CRISPR screening, T cell dysfunction score, immune-suppressive rejection score, and therapeutic response of ICB (Figure 6E and Supplementary Table 3). Therefore, the heterogeneity of immune cell infiltration in PC may serve as a novel indicator and has potential clinical implication in immunotherapy.

### Potential Predictive Biomarker for Chemotherapy and Targeted Therapy

**FIGURE 7:**
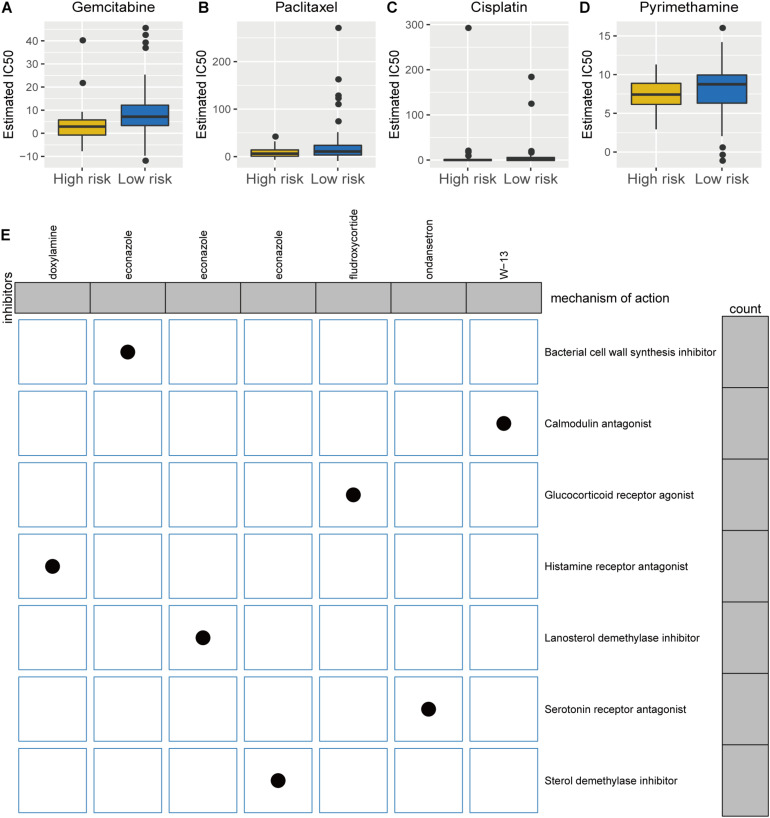
Differential chemotherapeutic responses and targeted traits of patients of high- and low-risk pancreatic cancer (PC). **(A–D)** Estimated IC_50_ for gemcitabine **(A)**, paclitaxel **(B)**, cisplatin **(C)**, and pyrimethamine **(D)** between high- and low-risk patients with PC. **(E)** Intrinsic mechanisms of candidate inhibitors targeted the established signature: Connectivity Map analysis.

Besides immunotherapy, chemotherapy and targeted therapy are currently two major adjuvant therapies in PC treatment (Bear et al., 2020; Christenson et al., 2020). Since chemotherapy is a classical and effective way in treating PC, we assessed the therapeutic responses of the two risk phenotypes to 138 chemotherapeutic agents. We put our predictive signature into the GDSC database for training. A significant difference in the estimated half inhibitor concentration (IC_50_) between the two phenotypes was observed, where patients in the high-risk group demonstrated high sensitivity to 48 types of representative or promising chemotherapeutic drugs (Supplementary Figure 2), like gemcitabine (*p* < 0.0001; Figure 7A), paclitaxel (*p* = 0.030; Figure 7B), cisplatin (*p* = 0.009; Figure 7C), and pyrimethamine (*p* = 0.018; Figure 7D). Next, analysis on the CMap approach was conducted and identified five of eight available candidate compounds/inhibitors that targeted the TP53-associated signature, including doxylamine, econazole, fuldroxycortide, ondansetron, and W-13. Using the mode-of-action (MoA) analysis, the aforementioned potential drugs are unveiled enriched in calmodulin antagonist, glucocorticoid receptor agonist, histamine receptor antagonist, lanosterol demethylase inhibitor, serotonin receptor antagonist, and sterol demethylase inhibitor (Figure 7E and Supplementary Table 4). Taken together, the established TP53-associated signature might provide guidance for selecting sensitive chemotherapeutic agents and developing individualized targeted drugs for PC.

## Discussion

Mutated in a wide range of cancer types and in over 70% of PC (Waddell et al., 2015; Kastenhuber and Lowe, 2017), tumor suppressor TP53 played a pivotal role in cellular stress response and acted as a tumor suppressor gene in PC (Kruiswijk et al., 2015; Kleeff et al., 2016). The past few years have witnessed tremendous efforts toward the development of promising candidate biomarkers in PC, among them the prognostic significance of TP53 mutation had been proposed (Grochola et al., 2011; Ormanns et al., 2014). Although the oncogenic role of TP53 mutation has been well documented, currently, there is a lack of a prognostic and therapeutically predictive biomarker based on the TP53 mutational status in PC. In the present study, we investigated the landscape and phenotypes of TP53 mutation in PC. We found that TP53 mutation played a prognostic and oncogenic role in PC, contributing to pathways of tumor growth and progression. We profiled a differentially expressed gene set affected by TP53 mutational status and generated a TP53-associated signature that could identify patients with poor OS, enhanced immune infiltration, and remarkable therapeutic sensitivity. Our results revealed promising value of the TP53-associated signature in clinical prognostic assessment. It could also be used to determine drug therapeutic strategy for patients who are not suitable candidates for surgery. To our knowledge, this study was the first to describe a novel model for prognostic assessment and therapeutic response based on the mutational status of driver genes in PC.

In this study, LASSO–Cox analyses were employed to construct a TP53-associated signature, composed of UCA1, SLC26A11, LINC01559, ARNT2, and TRIM67 genes. These five genes possessed the potential for individual target and may perform better in combination. These genes were proposed to play roles in regulating immune functions. Induced by hypoxia-inducible factor (HIF)-1α in the presence of hypoxia, UCA1 is dysregulated in tumors and plays a role in carcinogenesis (Chen et al., 2018). In PC, hypoxic exosome-mediated UCA1 could promote tumor angiogenesis and accelerate tumor growth *via* the miR-96-5P/AMOTL2/ERK1/2 axis and serve as a novel therapeutic target (Guo et al., 2020). Previous study reported that upregulated UCA1 promoted programmed death ligand 1 (PDL1) expression through the repression of miRNAs and contributed to immune escape in cancers (Wang C. J. et al., 2019). SLC26A11 is a chloride transporter facilitating acid secretion, with the localization of the vacuolar H + -ATPase in the A-intercalated cells of the kidney (Xu et al., 2011). TIDE analysis revealed that SLC26A11 is downregulated in the TP53-mutated PC and had remarkable association with immune-related processes, including interactions with cytotoxic T cells to affect patient prognoses, regulation of immunosuppressive cells that promoted T cell rejection, and ICB. Our results demonstrated the significance of SLC26A11 in immune-regulatory and oncogenic processes. In a previous study, a myriad of evidence has demonstrated the crucial roles of LINC01559 and ARNT2 in carcinogenesis and tumor progression. For example, LINC01559 could facilitate pancreatic tumor proliferation and migration through the regulation of Rubisco accumulation factor (RAF)1 overexpression and Yes-associated protein (YAP)-mediated pathway (Chen et al., 2020; Lou et al., 2020); located at the hub of transcription factor network, ARNT2 functions as a key component of oncogenic signature, contributing to cancer cell aggressiveness (Bogeas et al., 2018). However, whether immunological factors play critical roles in oncogenesis remain enigmatic, and our research revealed the immune-related oncogenic effects of LINC01559 and ARNT2 for the first time. Functioning as a transcriptional target bounded by p53 and crucial tumor suppressor, TRIM67 boosted apoptosis and p53-induced tumor growth suppression and improved chemotherapeutic responsiveness (Wang S. et al., 2019). Therefore, these five genes might play roles in cancers partly by affecting immune responses.

Further analyses suggested the accuracy, independence, and robustness of the TP53-associated signature in our study. We found that patients in the high-risk group had remarkably worse outcomes with the mean of AUC more than 0.75 in TCGA cohort and validated in the GSE28735, GSE62452, and GSE78229 cohorts. Moreover, this signature was proven to be an independent prognostic factor upon multivariable and stratified survival analyses of several clinical characteristics. Therefore, this TP53-associated signature has the potential to improve prognostic accuracy of traditional clinical factors and could serve as a promising tool for clinical use.

The tumor microenvironment mediated by epithelial–stromal cell interactions is emerging as a critical contributing factor of pancreatic cancer relapse and metastasis, impairing the effectiveness of chemotherapy and immunotherapy (Chronopoulos et al., 2016; Ren et al., 2018). Extensive studies on tumor microenvironment have suggested the pivotal role of immune cell infiltration in tumor dissemination, progression, metastasis, as well as immunotherapeutic response (Zeng et al., 2018; Zhang et al., 2020). Here, we investigated the characteristics of immune cell infiltration based on the TP53-associated signature and intrinsic traits related to the efficacy of cancer immunotherapy. High-risk PC patients tended to possess high proportions of macrophages M0 and resting NK cells and low proportions of naive B cells and CD8^+^ T cells. Tumor-associated macrophages (TAMs) are capable of promoting tumor growth and progression during almost all stages of cancers *via* the secretion of immunosuppressive factors like interleukin-10 (IL-10) (De Palma and Lewis, 2013). Associated with unfavorable prognosis, TAMs are also attractive targets due to their effect on immunotherapy, chemotherapy, and monoclonal targeted therapy (Noy and Pollard, 2014). TAM-secreted cytokines are known to weaken the anticancer effect of tumor-infiltrating lymphocytes (TLSs). CD8^+^ T cell is one of such TLSs whose abundance is linked to favorable prognosis and immunotherapeutic response (Vassilakopoulou et al., 2016). Pioneering studies have suggested that recruitment and reactivation of CD8^+^ T cell infiltration could be objectives of immunotherapies (Zhang et al., 2017). To meet this objective, intercellular interactions in tumor-infiltrating cells are more crucial than single-agent activity. For example, facilitating BAG3 blockade leads to higher infiltration of CD8^+^ T cells in PC possibly due to decreased secretion of TAM-derived factor (Iorio et al., 2018). Trafficking into pancreatic tumor microenvironment, endogenous CD8^+^ T cells reactivate recognition and destruction of neoplastic cells by the combination of programmed cell death protein 1 (PD-1) and C-X-C chemokine receptor type 4 (CXCR4) blockade as the basis for combination immunotherapy in PC (Seo et al., 2019). We suggested that the induction of immunosuppressive microenvironment likely underlies poor prognosis and treatment-refractory nature of the high-risk patients.

The optimal treatment strategy of locally advanced or metastatic PC remains challenged, given the absence of selecting population most likely to benefit from available standard chemotherapeutic regimens. Analysis on GDSC showed the difference of chemotherapeutic sensitivity between TP53-associated risk phenotypes. High-risk patients are more sensitive to the 48 chemo drugs, including gemcitabine and paclitaxel. Recently, pioneering investigation endorsed the activity and safety of nab-paclitaxel plus gemcitabine (AG) as the first-line chemotherapy in localized PC (Perri et al., 2020; Philip et al., 2020). In addition to AG, some other chemotherapeutic agents, like istiratumab (NCT02399137) (Kundranda et al., 2020), capecitabine and cisplatin (NCT01730222) (Reni et al., 2018), were shown to improve the treatment efficacy on metastatic PC. Besides, high-risk patients demonstrated high sensitivity in some novel agents in cancer treatment, such as ABT-263 (Navitoclax) and pyrimethamine, providing great insight into novel chemo drugs for PC treatment. Moreover, five potential inhibitors that target TP53-associated signature were screened out according to CMap database and MoA analysis. Previous studies have seldom reported the application of these drugs in the treatment of PC, with the exception of doxylamine [histamine receptor antagonist (HRA)]. A similar analysis has previously identified doxylamine as a potential drug that targeted lncRNA in non-homologous end joining pathway I in early stage pancreatic ductal adenocarcinoma based on genomic expression profile (Shang et al., 2020). Interactions between HRA and metformin may play a crucial role in inhibiting pancreatic carcinogenesis (Broadhurst and Hart, 2018). The present study suggested doxylamine and HRA as promising drugs for PC. However, the mechanism and effectiveness of specified agents for treatment of PC warrant further investigation and elucidation.

In summary, we constructed and validated a genomic signature based on TP53 mutational status in PC. The TP53-associated signature can be used for prognostic stratification and can reflect immune cell infiltration. It can also serve as a multifaceted therapeutic indicator.

## Data Availability Statement

All data used in this work can be acquired from the TCGA (https://portal.gdc.cancer.gov/repository), GEO (https://www.ncbi.nlm.nih.gov/geo), GDSC (https://www.cancerrxgene.org), and TIDE (http://tide.dfci.harvard.edu). The accession number(s) can be found in the article/Supplementary Material.

## Author Contributions

FZ, MY, and YL designed this work. FZ, WZ, and KH integrated the data and conducted the analyses. FZ and HL wrote this manuscript. YL, MY, and KH edited and revised the manuscript. All authors approved this manuscript.

## Conflict of Interest

The authors declare that the research was conducted in the absence of any commercial or financial relationships that could be construed as a potential conflict of interest.

## Footnotes

1 https://portal.gdc.cancer.gov/repository

2 https://www.ncbi.nlm.nih.gov/geo

3 http://tide.dfci.harvard.edu

4 https://www.cancerrxgene.org
