# Supplementary material for: TP53 Mutational Status-Based Genomic Signature for Prognosis and Predicting Therapeutic Response in Pancreatic Cancer
Source: Front Cell Dev Biol. 2021 May 26;9:665265. doi: 10.3389/fcell.2021.665265 (PMC8187932; doi:10.3389/fcell.2021.665265)
Supplement: Supplementary file 5 [file Data_Sheet_1.docx]

# Supplementary Figures and Tables

**Supplementary Figure 1.** (A) Time-dependent ROC analysis of TP53-associated signature, TP53 mutation and clinicopathological factors. (B) Conditional survival analysis in the combined TCGA, GSE28735, GSE62452, GSE78229 cohorts.

**Supplementary Figure 2.** Estimated IC50 of 48 chemotherapeutic agents between high and low risk patients with PC.

**Supplementary Table 1.** DEGs between TP53-mutated and TP53-wild PC.

**Supplementary Table 2.** Correlations between five genes involved in the TP53-associated signature.

**Supplementary Table 3.** The immunosuppressive role of five genes demonstrated by TIDE database involved in CRISPR screening, T cell dysfunction score, immune-suppressive rejection score and therapeutic response of ICB.

**Supplementary Table 4.** Candidate compounds/inhibitors that targeted the TP53-associated signature derived by CMap and MoA analysis.
