# Supplementary figures and images for: TP53 Mutational Status-Based Genomic Signature for Prognosis and Predicting Therapeutic Response in Pancreatic Cancer
Source: Front Cell Dev Biol. 2021 May 26;9:665265. doi: 10.3389/fcell.2021.665265 (PMC8187932; doi:10.3389/fcell.2021.665265)

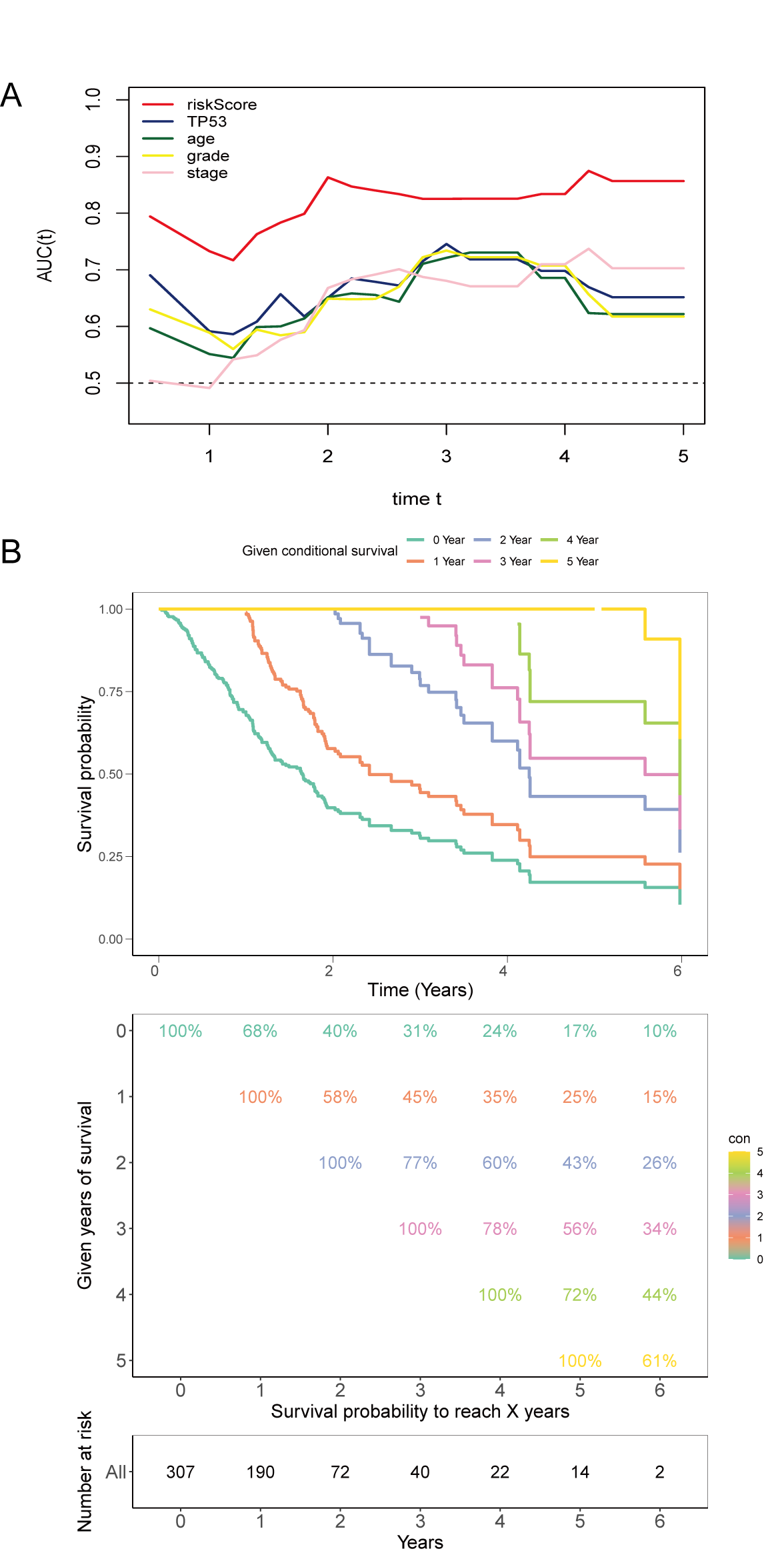

Supplement: Supplementary file 6 [file Image_1.TIF]

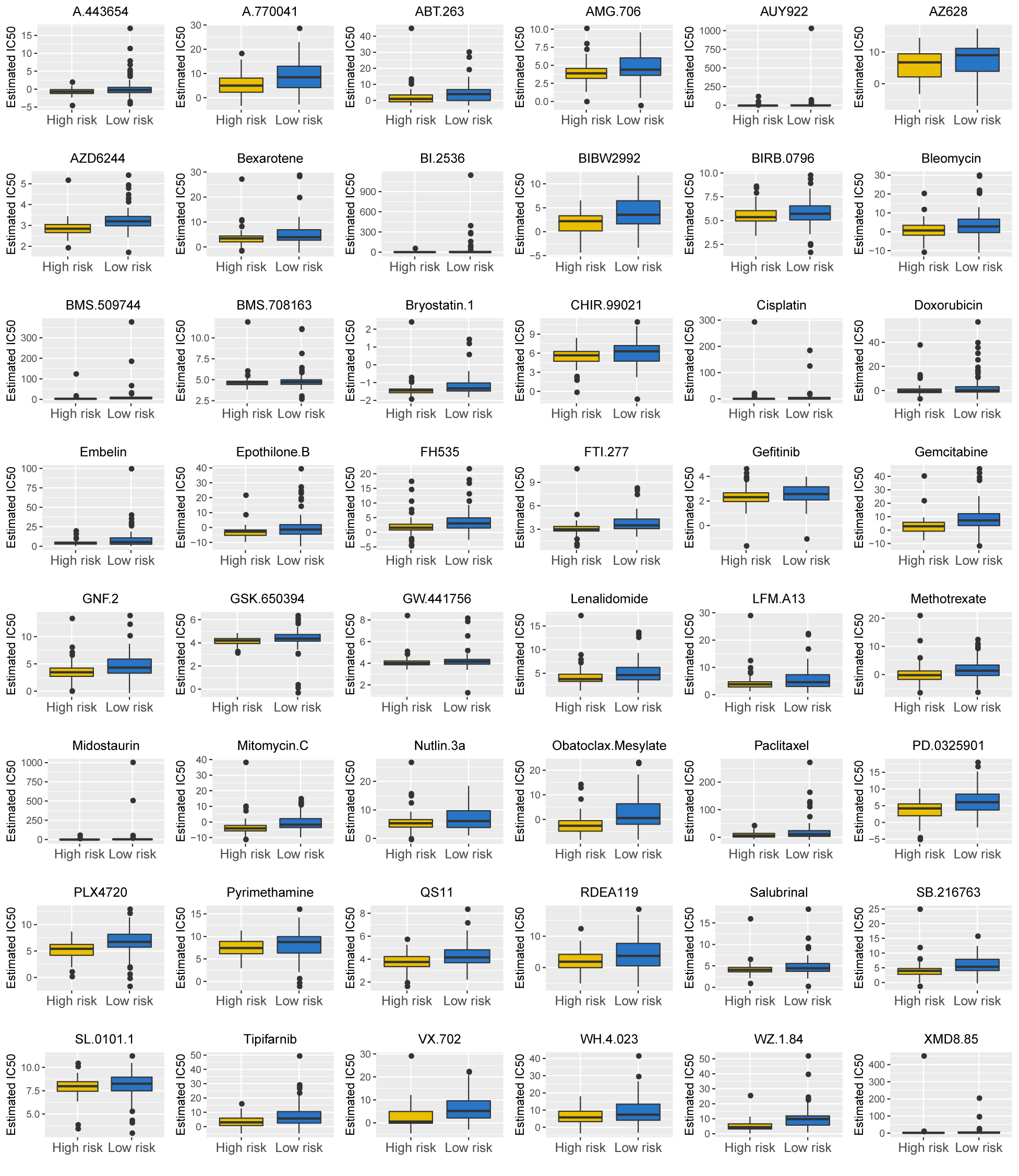

Supplement: Supplementary file 7 [file Image_2.TIF]
